# Transoral Videolaryngoscopic Surgery for an Undifferentiated Pleomorphic Sarcoma of the Tongue Base: A Case Report

**DOI:** 10.3390/reports8020058

**Published:** 2025-04-28

**Authors:** Takayuki Taruya, Takao Hamamoto, Tsutomu Ueda, Nobuyuki Chikuie, Sachio Takeno

**Affiliations:** Department of Otorhinolaryngology, Head and Neck Surgery, Hiroshima University Hospital, 1-2-3, Kasumi, Minami-ku, Hiroshima 734-8551, Japan; takao0320@hiroshima-u.ac.jp (T.H.); uedatsu@hiroshima-u.ac.jp (T.U.); housejak@hiroshima-u.ac.jp (N.C.); takeno@hiroshima-u.ac.jp (S.T.)

**Keywords:** undifferentiated pleomorphic sarcoma, malignant fibrous histiocytoma, undifferentiated sarcoma, tongue base, oropharynx, transoral endoscopic surgery, transoral videolaryngoscopic surgery

## Abstract

**Background and Clinical Significance:** Undifferentiated pleomorphic sarcoma (UPS) is a highly malignant soft tissue tumor formerly known as malignant fibrous histiocytoma. In the fifth edition of the WHO classification (2020), UPS is classified as an undifferentiated/unclassifiable sarcoma diagnosed via exclusion. While UPS commonly occurs in the extremities, its incidence in the head and neck region is rare (3%), with only a few reported cases in the oropharynx. Surgical resection is the primary treatment; however, tumors at the tongue base pose significant challenges due to the complex anatomy and the presence of critical neurovascular structures. This case highlights a rare instance of tongue-base UPS successfully treated with transoral videolaryngoscopic surgery (TOVS), demonstrating its feasibility as a minimally invasive approach. **Case Presentation:** A 68-year-old male presented with pharyngeal discomfort, dysphagia, and nocturnal dyspnea. Clinical examination revealed a pedunculated tumor originating from the left tongue base, occupying the pharyngeal cavity. Imaging studies showed a 5 cm mass without lymph node metastasis. A biopsy confirmed UPS (cT3N0M0). Given the tumor’s characteristics, TOVS was performed using an FK-WO TORS laryngo-pharyngoscope retractor. The tumor was resected with a ≥10 mm margin, achieving complete histological resection. The patient’s dyspnea resolved immediately, and oral intake resumed the next day. No adjuvant radiotherapy was administered, and no recurrence was observed for 50 months. **Conclusions:** This is the first reported case of UPS of the tongue base successfully resected using TOVS. This minimally invasive approach provides a safe and effective alternative for managing oropharyngeal UPS.

## 1. Introduction and Clinical Significance

Undifferentiated pleomorphic sarcoma (UPS) is a highly malignant soft tissue tumor that was previously referred to as malignant fibrous histiocytoma (MFH), but in the fifth edition of the WHO classification (2020) [1] it is classified as a subtype of undifferentiated/unclassifiable sarcoma for which no clear differentiation tendency can be identified and diagnosed via exclusion [2]. UPS typically occurs in the extremities and rarely in the head and neck region (3%) [2]. Among head and neck sites, the most common subsites are the parotid gland and neck [3]. Only a few cases of UPS in the oropharynx have been reported [4,5]. The initial surgical resection for sarcoma in the head and neck region should aim to be as radical as possible in order to reduce the chance of local recurrence and to improve outcomes [6,7,8]. However, surgical resection at the tongue base remains challenging due to its complex anatomy and the presence of critical neurovascular structures, including the hypoglossal nerve and lingual artery [9]. In recent years, advancements in medical technology have facilitated the adoption of various surgical approaches, including conventional open surgery and transoral endoscopic procedures [9,10,11,12]. We report a rare case of UPS at the tongue base successfully resected using transoral videolaryngoscopic surgery (TOVS).

Clinical Significance: This is the first transoral surgery report for a rare undifferentiated pleomorphic sarcoma originating in the oropharynx.

## 2. Case Presentation

**Figure 1:**
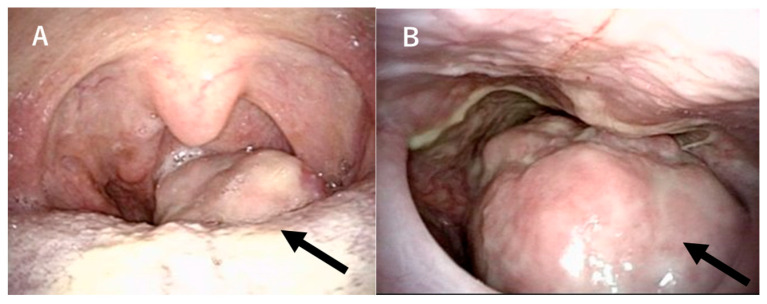
Tongue-base tumor findings with transoral endoscopy (**A**) and transnasal endoscopy (**B**). A tumor with a smooth surface protruding from the left tongue base occupies the oropharynx (black arrow).

**Figure 2:**
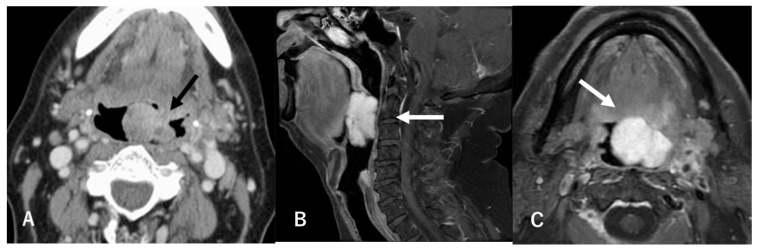
Imaging findings of the tongue-base mass. Contrast-enhanced CT scans showing a 5 cm protruding mass (black arrow) in the left tongue base (**A**). Contrast-enhanced MRI showing a hyperintense protruding mass with a well-defined shadow in the left tongue base (white arrow). Sagittal section (**B**). Transverse section (**C**).

A 68-year-old male presented to our hospital with pharyngeal discomfort and dysphagia that had worsened over the past two months, along with nocturnal dyspnea that started a month ago. On clinical examination, transoral and transnasal endoscopic evaluation was performed. Transoral (Figure 1A) and transnasal (Figure 1B) endoscopy revealed a pedunculated tumor with a smooth surface arising from the left half of the tongue base, protruding into and occupying a large portion of the oropharynx (black arrow). The mass was visibly protruding into the oropharyngeal space, leading to partial airway obstruction, which explained the patient’s nocturnal breathing difficulty. Endoscopic inspection revealed no signs of overt bleeding or ulceration. Contrast-enhanced CT scans showed a 5 cm tumor with no evidence of lymph node metastasis or distant metastasis (Figure 2A). Contrast-enhanced T1-weighted magnetic resonance imaging revealed a hyperintense protruding mass with a well-defined shadow in the left tongue base (Figure 2B,C). It exhibited a homogeneously low intensity in T1WI and was hyperintense in fat-suppression T2WI. A transoral biopsy was performed under local anesthesia using 1% lidocaine with epinephrine.

Histologically, spindle-shaped or polygonal tumor cells with atypia showed diffuse proliferation in the subepithelial tissue. The tumor cells varied in size and exhibited marked pleomorphism. Immunohistochemically, the tumor cells were positive for vimentin, a mesenchymal marker, but negative for epithelial markers (CK AE1/AE3, CK7, and CK20), myogenic markers (desmin and α-smooth muscle actin), neurogenic markers (CD56 and S-100), lymphoid markers (CD45 and CD30), and endothelial markers (CD31). Other lineage-specific markers, including CD68, ALK-1, CD99, MDM2, CDK4, and MPO, were also negative. These findings provided no definitive evidence of differentiation into a specific cellular lineage.

The histopathological and immunohistochemical features were consistent with a diagnosis of undifferentiated pleomorphic sarcoma (UPS). Based on the AJCC’s staging system, eighth edition, the tumor was classified as cT3N0M0.

Given the rarity of this tumor at the tongue base and the progressive nature of the symptoms, a total resection was planned for both diagnostic and therapeutic purposes.

**Figure 3:**
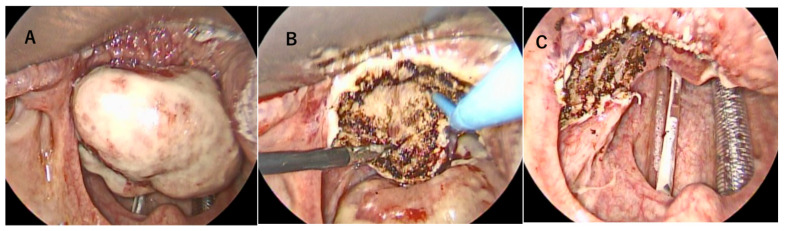
Intraoperative findings. Before incision (**A**). The tongue base being cut using a needle-shaped electrosurgical knife (**B**). The completely resected tumor (**C**).

**Figure 4:**
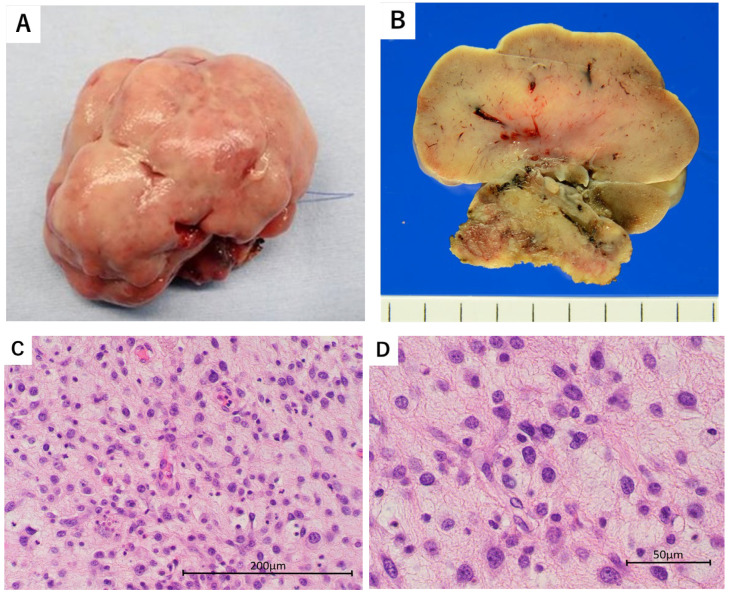
(**A**) The entire resected tumor. (**B**) A cross-section of the resected tumor. Histologically diffuse proliferation of spindle-shaped or polygonal tumor cells with atypia, exhibiting irregular size and polymorphism. The nuclear chromatin is darkly stained, and a few fissionable nuclei are seen. Low-power field (**C**). High-power field (**D**).

Transoral videolaryngoscopic surgery (TOVS) was performed. Despite the tumor occupying the pharyngeal cavity, nasotracheal intubation was successfully performed by pulling the tongue forward and expanding the larynx. The surgical field was expanded using an FK-WO TORS laryngo-pharyngoscope retractor (Olympus, Hachioji City, Tokyo). A rigid endoscope was inserted, and the oropharyngeal lesion was clearly visualized. Tumor resection was performed under endoscopic guidance using a disposable needle-shaped electrosurgical knife. The tumor was grasped and dissected using CLICKLINE Maryland forceps (Karl Storz, Tuttlingen, Germany), allowing for precise manipulation in the confined surgical field. The left half of the tongue base and the tumor were resected with a margin of more than 10 mm (Figure 3). Histologically complete resection was achieved. Following tumor removal, the left lingual artery was identified and ligated orally to ensure hemostasis. Excisional specimens revealed a smooth-surfaced, grayish-white tumor measuring 5 cm (Figure 4A,B). Histopathological examination demonstrated undifferentiated pleomorphic sarcoma involving the oropharynx. The tumor exhibited diffuse proliferation of spindle-shaped or polygonal cells with marked atypia. The tumor cells varied in size and shape, showing significant pleomorphism, hyperchromatic nuclei, and numerous mitotic figures (Figure 4C,D).

The immunohistochemical findings were consistent with those observed in the preoperative biopsy: the tumor cells were positive for vimentin, a mesenchymal marker, but negative for epithelial markers (CK AE1/AE3, CK7, and CK20), myogenic markers (desmin and α-smooth muscle actin), neurogenic markers (CD56 and S-100), lymphoid markers (CD45 and CD30), and endothelial markers (CD31). Other lineage-specific markers, including CD68, ALK-1, CD99, MDM2, CDK4, and MPO, were also negative, indicating no evidence of differentiation toward a specific cellular lineage. The Ki-67 labeling index was approximately 70%, supporting the tumor’s high proliferative activity.

According to the FNCLCC grading system, the tumor received a differentiation score of 3, a mitotic count score of 3, and a necrosis score of 0, resulting in histological grade 3.

Collectively, these histopathological and immunohistochemical features support the diagnosis of undifferentiated pleomorphic sarcoma. After surgery, dyspnea disappeared immediately, and oral intake became possible the next day. The resection margins were negative, and although adjuvant postoperative radiotherapy was recommended, the patient declined due to personal preference. No recurrence was observed over a 50-month follow-up period.

## 3. Discussion

Previous reports indicate that UPS most commonly occurs in the soft tissues of the extremities and retroperitoneum, with an incidence of approximately 3% in the head and neck region [2]. UPS in the head and neck region may also develop as radiation-induced sarcoma following radiotherapy for head and neck cancer [13]. In a report of 95 cases of head and neck UPS, lesions after previous radiotherapy were reported to occur in 23% of cases [3], and radiation-induced sarcomas are considered to have a significantly poorer prognosis than sarcomas that occur independently of radiation exposure [13]. In head and neck UPS, the parotid gland and neck are the most common sites [3]. Reports of primary tumors of the oropharynx are rare, and to the best of our knowledge there are only two previous reports of this type of tumor [4,5].

UPS is often characterized by rapid growth, but it lacks distinctive clinical features that differentiate it from other sarcomas [1]. In this case, however, the patient had dysphagia and nocturnal dyspnea due to the 5 cm tumor occupying the pharyngeal cavity. There have been reports of a case of oropharyngeal UPS that developed 10 years after radiotherapy for tongue-base cancer, where symptoms of airway obstruction necessitated emergency tracheotomy due to the rapidly growing tumor [4], and a case of dysphagia due to a 21 mm UPS that developed on the posterior wall of the oropharynx [5].

The first choice of treatment for head and neck UPS is surgical resection with a sufficient safety margin, and positive margins are thought to be associated with a decrease in survival rate [3,14]. As with most high-grade sarcomas, the R0 resection of UPS is typically carried out with a 1 cm margin of normal tissue or by removing the adjacent fascial margin [8]. In a report of 207 cases of UPS involving the entire area, the cumulative local recurrence rate was 3% for resection margins of 10 mm or more compared to 14% and 25% for margins of 0.1–9.9 mm and 0 mm (*p* = 0.026), respectively [15]. In a report of 54 cases of head and neck UPS, the cumulative local recurrence rate for marginal resection and wide-area resection was 86% and 66%, respectively, while the rate for radical resection (resection to the boundary of the adjacent normal fascia surface or adjacent healthy organ/tissue) was 27% (*p* = 0.01) [6].

In the head and neck region, surgical resection is often more challenging than in other anatomical areas due to the close proximity of critical structures such as the eyes, brain, and major neurovascular bundles [7]. However, in the present case, the lesion was pedunculated and located at the tongue base, which allowed for adequate endoscopic visualization and the achievement of a 1 cm surgical margin.

Traditionally, tongue-base tumors have been resected through transcervical or transmandibular approaches. These methods carry a significant risk of injury to the lower cranial nerves and surrounding musculature before reaching the tumor. Additionally, there is a potential risk of damage to the mandible and teeth [16]. Conventional transoral surgery has also been limited by poor visualization and restricted instrument maneuverability under standard lighting conditions. As a result, only small tumors located in relatively accessible sites such as the tonsils, soft palate, or posterior pharyngeal wall have historically been considered suitable for transoral resection.

To address these limitations, endoscopic-assisted transoral approaches—including transoral robotic surgery (TORS) and transoral videolaryngoscopic surgery (TOVS)—have recently been introduced for the resection of tumors located in anatomically challenging areas such as the tongue base [9,11,12]. These techniques offer improved visualization and instrument control, enabling safer and more effective tumor removal. The transoral approach is particularly suitable for T1, T2, and select T3 lesions that exhibit exophytic growth patterns. In contrast, en bloc resection of infiltrative tumors involving structures such as thyroid cartilage, hyoid bone, or cricoid cartilage remains technically demanding and is generally not indicated [11]. In this case, the lesion was over 4 cm in size and diagnosed as T3N0M0, but it was a broad-based protruding lesion that infiltrated the left half of the tongue base; thus, it was indicated for transoral resection. At the time of surgery, TORS was not covered by health insurance in Japan; we subsequently used the method of Tomifuji et al. [11] to perform the resection using TOVS. The base of the tumor could be clearly observed by expanding the larynx with the FK-WO TORS laryngo-pharyngoscope retractor; hence, there were no difficulties in performing the resection using TOVS. Compared to conventional transcervical approaches, endoscopic-assisted transoral resection has been associated with reduced rates of gastric tube placement and tracheostomy [17].

In this case, the patient had difficulty breathing at night due to the narrowing airway before the resection, but after the resection the airway was cleared; thus, the tracheal tube could be removed after the surgery, and the breathing difficulty symptoms disappeared immediately after the surgery. In addition, oral intake was possible from the next day, making it a minimally invasive surgery.

In a randomized controlled trial comparing surgery alone and perioperative adjuvant chemotherapy for malignant soft tissue tumors, the only study that showed the superiority of perioperative chemotherapy in overall survival was a study using doxorubicin and ifosfamide for spindle cell sarcoma [18].

In recent years, the application of perioperative radiation therapy has been increasingly investigated as part of multidisciplinary treatment strategies for high-grade soft tissue sarcomas. Decisions regarding the use of adjuvant radiation therapy are generally based on evidence from randomized or phase 3 clinical trials, particularly those evaluating limb-sparing surgery in combination with radiation therapy. According to Yang et al., postoperative radiation therapy significantly reduced the cumulative local recurrence rate (*p* = 0.0028), although no significant difference in overall survival was observed [19]. In a study involving 109 cases of UPS at various anatomical sites, postoperative radiotherapy was shown to be effective in improving local control in patients with positive resection margins [20]. However, in cases with negative surgical margins, other studies have suggested that the margin status itself is the most critical factor in local control, and that adjuvant radiotherapy may be omitted through careful patient selection [21].

In the present case, a pathological margin of at least 1 cm was achieved. Based on this finding, a multidisciplinary team (MDT) discussion was held, and adjuvant postoperative radiotherapy was recommended. However, the patient declined radiotherapy due to severe physical deconditioning caused by two months of dysphagia prior to treatment. In respect of the patient’s wishes, postoperative radiation therapy was not administered. As a result, the patient has remained free of local recurrence for 50 months, and local disease control has been successfully achieved with surgery alone.

To further contextualize the treatment outcomes of UPS in this region, we conducted a review of 11 reported cases of UPS arising in the tongue, oral floor, or oropharynx treated since 1990, including the present case (Table 1). Although the number of cases is limited, long-term survival of 18 months or more was observed only in patients who had no prior radiation history or who underwent surgery as the primary treatment with negative surgical margins—either wide or ≥1 cm. Among the seven surgically treated patients, only two received postoperative radiotherapy. Importantly, three patients, including our present case, remained recurrence-free for over 18 months with surgery alone. These findings underscore the critical importance of achieving adequate surgical margins in the management of UPS in these anatomical regions.

It has been reported that 44% of patients with primary head and neck UPS develop distant metastases [3], highlighting its high propensity for distant spread. There is no established standard drug therapy for recurrent or metastatic UPS, and the same regimen as other recurrent or metastatic soft tissue sarcomas must be used. Doxorubicin monotherapy is considered the current standard treatment [30]. In a phase 2 trial using pembrolizumab for soft tissue sarcoma, the response rate was 18% in 40 cases of soft tissue sarcoma that were unresectable or had distant metastases; moreover, the response rate was 40% in 10 cases of UPS, suggesting that pembrolizumab may be more effective than conventional chemotherapy [31]. The present case exemplifies the potential for long-term local control of oropharyngeal UPS through transoral resection alone, without adjuvant therapy, when an adequate surgical margin can be achieved. Despite the patient’s preoperative physical decline due to prolonged dysphagia, the minimally invasive nature of the transoral approach enabled safe and effective treatment, leading to a favorable oncologic and functional outcome. This case reinforces the importance of individualized treatment planning and supports the role of transoral surgery as a viable option in selected patients with UPS of the tongue base.

## 4. Conclusions

Transoral videolaryngoscopic surgery (TOVS) represents a feasible and effective minimally invasive option for the treatment of undifferentiated pleomorphic sarcoma (UPS) of the tongue base.

In the present case, TOVS enabled safe and complete tumor resection with oncologically adequate margins, while preserving critical anatomical structures.

The patient’s favorable postoperative recovery and long-term recurrence-free survival underscore the potential utility of this approach in selected patients with oropharyngeal UPS.

## Figures and Tables

**Table 1:** Case data.

Article	Age; Gender	Tumor Location	Long Diameter (cm)	Prior Radiation	Primary Treatment	Resection Margin	Adjuvant Therapy	Follow-Up
Lin et al., 1994 [22]	57; Male	Tongue	ND	2.5 y ago	Surgical excision	ND	No	6 m rec; 26 m DOD
Zapater et al., 1995 [23]	71; Male	Tongue	4	No	RT	NA	No	8 m rec; 9 m DOD
Chen et al., 2001 [24]	16; Female	Tongue	2.5	No	Surgical excision	Negative	No	18 m NER
Wiesmiller et al., 2003 [25]	79; Male	Tongue	3	5 y ago	Surgical excision	ND	No	6 m NER
Rapidis et al., 2005 [26]	24; Male	Tongue	2.6	No	Surgical excision	1 cm	No	18 m NER
Alfredo et al., 2008 [27]	56; Male	Oral floor	4.5	No	Surgical excision	Wide margin	RT	25 m NER
Wu et al., 2022 [28]	49; Male	Oral floor	5	No	Surgical excision	2 cm	RT CT	26 m NER
Sharma et al., 2023 [29]	50; Female	Oral floor	4.5	No	Surgical excision	Wide margin	No	ND
Sadati et al., 2004 [4]	60; Male	Lateral oropharyngeal wall	ND	10 y ago	No (best supportive care)	NA	No	2 m DOD
Dekanić et al., 2021 [5]	83; Male	Posterior oropharyngeal wall	2.5	No	Surgical excision	ND	No	ND
Taruya et al., 2025	68; Male	Tongue base	5	No	Surgical excision	1 cm	No	50 m NER

**Abbreviations:** ND = not described; NA = not applicable; DOD = died of disease; NER = no evidence of recurrence; RT = radiotherapy; CT = chemotherapy.

## Data Availability

Data are contained within the article, further inquiries can be directed to the corresponding author.

## Abbreviations

The following abbreviations are used in this manuscript:

UPS	Undifferentiated pleomorphic sarcoma
MFH	Malignant fibrous histiocytoma
TOVS	Transoral videolaryngoscopic surgery
TORS	Transoral robotic surgery
